# Components of Rhizome Extract of *Cnidium officinale* Makino and Their *In vitro* Biological Effects 

**DOI:** 10.3390/molecules16108833

**Published:** 2011-10-21

**Authors:** Ki-Eun Bae, Young-Woong Choi, Sang-Tae Kim, Young-Kyoon Kim

**Affiliations:** Department of Forest Products and Biotechnology, College of Forest Sciences, Kookmin University, Seoul, 136-702, Korea

**Keywords:** nitric oxide synthase (NOS), cyclooxygenase-2 (COX-2), Bax, p53, Bcl-2, falcarindiol

## Abstract

The anti-inflammatory and anticancer activities of a methanol extract of the rhizome of *Cnidium officinale* were investigated. Four compounds, namely falcarindiol (**1**), 6-hydroxy-7-methoxy-dihydroligustilide (**2**), ligustilidiol (**3**), and senkyunolide H (**4**) were isolated from the extract of the rhizome of *Cnidium officinale* and their structures were elucidated by analysis of their spectroscopic data and by comparison with previously reported data. These compounds showed anti-inflammatory activities, measured as inhibition of nitric oxide (NO) release in lipopolysaccharide (LPS)-stimulated RAW 264.7 macrophage cells, with IC_50_ values of 4.31 ± 5.22, 152.95 ± 4.23, 72.78 ± 5.13, and 173.42 ± 3.22 μM, respectively. They also inhibited inducible nitric oxide synthase (iNOS) and cyclooxygenase-2 (COX-2) mRNA expression induced by LPS. Among these compounds, falcarindiol (**1**) was found to have anti-proliferative effect against MCF-7 human breast cancer cells by induction of a G_0_/G_1_ cell cycle block of the cells, with an IC_50_ value of 35.67 μM. Typical apoptotic effects were observed by phase contrast microscopy and were also exhibited in fluorescence microscopy with Hoechst 33342 staining. In addition, falcarindiol induced apoptosis through strongly increased mRNA expression of Bax and p53, and slightly reduced Bcl-2 mRNA levels in a dose dependent manner. This study suggested that *C. officinale* extract and its components would be valuable candidates in therapeutic applications for anti-inflammatory and anti-cancer agents.

## 1. Introduction

The medicinal plant *Cnidium officinale* Makino, called “Chunkung” in Korea, has been used in Asia as a natural drug for a long time. Chunkung is a perennial plant of the family Umbelliferae and is an important medicines used to fight diverse diseases [1,2]. This plant contains a variety of volatile phthalide derivatives which have been shown to have diverse pharmacological activities, including sedative, anti-anaemia, anti-fungal, smooth muscle relaxing, and anti-complementary properties. Furthermore, *C. officinale *was previously studied for its blood circulation and inflammatory disease regulatory properties [3,4]. This plant is also one of the major herbs used in oriental medicine to treat pain, inflammation and menstrual disturbances. Higashi *et al*. suggested that *C. officinale* may induce the relaxation or inactivation of blood congestion and inflammation and cause itching based on the promotion of blood circulation in inflammatory diseases [5]. In addition, other authors have also reported the anti-tumor and antimetastatic activities of *C. officinale *in ddY mice and hepatic carcinoma cells, respectively [6,7]. Therefore, studies of *C. officinale* have focused on a number of potential uses in various health-related areas including food processing, pharmaceuticals, and cosmetics industries [1].

Reactive oxygen species (ROS) are unstable and react readily with a wide range of biological substrates [8,9]. The overproduction of ROS disrupts cellular functions via denaturation of proteins, peroxidation of lipids, and oxidative damages of DNA, *etc*., leading to various chronic health problems such as inflammation, aging, and cancers. Therefore, many studies have suggested that agents with protective ability against ROS may be therapeutically useful in the treatment of these diseases [10,11].

Although nitric oxide (NO), as a reactive nitrogen species (RNS), plays an important role in host protection against some pathogenic microorganisms [12,13], increased levels of NO derived from inducible nitric oxide synthase (iNOS) activity during inflammatory responses, on the other hand, would result in the formation of peroxynitrite after reaction with oxygen free radicals. This cytotoxic species is involved in vasodilatation and tissue damage that mediate directly or indirectly malignant cell transformation [14]. Abnormal activation of pro-inflammatory mediators such as iNOS, cylooxygenase-2 (COX-2), and cytokines (interleukin-1, tumor necrosis factor-α and interferon-γ), induces various harmful responses including tissue injury and septic shock. Thus, the inhibition of NO production by suppressing iNOS expression is an important target in the treatment of inflammatory diseases.

Both ROS and RNS generated by activated inflammatory cells in response to various pro-inflammatory stimuli have another function as chemical effectors in inflammation-driven carcinogenesis [15]. Thus, certain forms of chronic inflammation can initiate tumorigenesis in the inflamed tissue and subsequent DNA damage leading to the activation of oncogenes and/or the inactivation of tumor suppressor genes [16].

Despite many therapeutic advances in cancer treatment [17], the death rate from the disease is still unacceptably high [18]. There has been a focus on a mechanistic approach for the development of chemotherapeutic cancer drugs by targeting the induction of apoptotic cell death since apoptosis, or programmed cell death, is an essential event that plays an important role in the organism’s development and its body cell homeostasis. Therefore, the search for agents capable of modulating the apoptosis pathway is currently an active area of investigation. In connection with our search for bioactive natural products from medicinal plants with potential therapeutic applications, the rhizome extract of *C. officinale* was investigated for its anti-inflammatory and anticancer activities. Four compounds, namely falcarindiol (**1**); 6-hydroxy-7-methoxydihydroligustilide (**2**); ligustilidiol (**3**); and senkyunolide H (**4**) were isolated from the extract of the rizhome of *Cnidium officinale*. We reporte here the chemical structures of these products and their biological properties.

## 2. Results and Discussion

### 2.1. Identification of Compounds

#### 2.1.1. Falcarindiol

**Figure 1:**
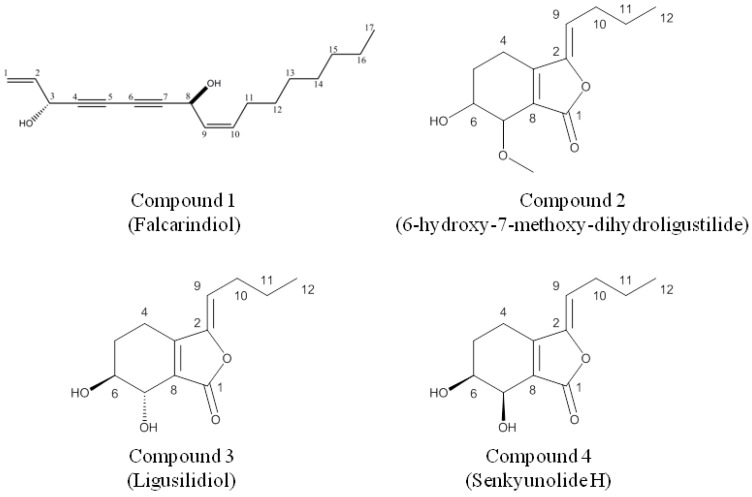
Chemical structures of isolated compounds.

The ^1^H spectrum exhibited an allylic alcohol moiety at δ 5.91 (1H, m, *J* = 17.1, 5.1 Hz), δ 5.46 (2H, m), 5.25 (2H, m) and δ 4.94 (1H, d, *J* = 5.1 Hz), two vinyl protons at δ 5.60 (1H, m, *J* = 10.5, 6.9 Hz) and δ 5.51 (1H, m, *J* = 10.5, 8.1 Hz) corresponding to a *cis* double bonds at Δ^9,10^, a hydroxylmethine protons at δ 5.20 (1H, d, *J* = 8.1 Hz), a triplet peak at δ 0.88 (3H, t, *J* = 6.7 Hz) due to a methyl group (H-17), and six methylene groups between δ 1.240–2.105. In particular, the four quaternary carbons at δ_C_ = 68.9, 70.5, 78.5 and 80.1 ppm indicated the presence of two triple bonds. Based on these data and the reference [19], compound 1 was identified as falcarindiol, [heptadeca-1,9(*cis*)-diene-4,6-diyne-3,8-ol] (Figure 1).

#### 2.1.2. (*Z*)-6-Hydroxy-7-methoxy-dihydroligustilide

Signals obtained from ^1^H and ^13^C-NMR spectra led to the identification of consisting of a butylidene side chain [δ 0.87 (3H, t, *J *= 7.2 Hz), H-12; δ 1.42 (2H, m, *J* = 7.2, 7.5 Hz), H-11; δ 2.27 (2H, q, *J* = 7.5, 7.8 Hz), H-10; and δ 5.24 (1H, t, *J* = 7.8 Hz), H-9], a methoxyl group (δ 3.45 (3H, s), a hydroxylmethine and a methoxylmethine protons (δ 3.87 and δ 4.09, H-6 and H7), as well as two methylene groups (δ 1.89 and δ 2.43, H-4 and H-5). By comparison with the literatures [20,21], compound 2 was identified as (*Z*)-6-hydroxyl-7-methoxy-dihydroligustilide (Figure 1).

#### 2.1.3. Ligustilidiol and Senkyunolide H

The ^1^H-NMR spectra of compounds identified as *trans-* and *cis*-*Z*-2-butylidene-4,5,6,7-tetrahydro-6,7-dihydrox1y-(3*H*)-isobenzofuranone, compound 3 and compound 4, respectively, were consistent with those reported for the *Z* isomers [19,20,21]. The carbon skeleton structure was also confirmed by a close similarity with the published ^13^C-NMR spectra for the *Z*-isomers. Comparison with published data for the *Z* isomers of compound 3 and 4 enabled the *trans* and *cis* stereochemistry of their structure to be determined [20]. The stereochemistry of the butylidene side-chain was determined by considering the chemical shifts of the olefinic proton. This have been reported as δ 5.5 and δ 5.3 for *E*- and *Z*-butylidenephthalide, respectively, and as δ 5.46 and δ 5.25 for *E*- and *Z*-ligustilide, respectively [20]. The observed chemical shift of the olefinic protons in compound 3 and compound 4 of δ 5.29 (1H, t, *J* = 7.8 Hz) indicated that the butylidene side-chain in these compounds has the *Z* configuration in the position [19,20,21]. The identification of a butylidene side chain [δ 0.88 (3H, t, *J *= 7.2 Hz), H-12; δ 1.42 (2H, m, *J* = 7.2, 7.5 Hz), H-11; δ 2.27 (2H, q, *J* = 7.5, 7.8 Hz), H-10; and δ 5.24 (1H, t, *J* = 7.8 Hz), H-9] and glycol group in the cyclohexene ring and their vicinal hydroxymethine protons appeared at δ 3.90 (1H, m) and 3.39 (1H, d, *J *= 5.7 Hz) in senkyunolide H, also clearly confirmed by comparison with the reference data [19,20,21] (Figure 1).

### 2.2. Measurement of Anti-Inflammation Activity

#### 2.2.1. Assessment of Cell Cytotoxicity

**Figure 2:**
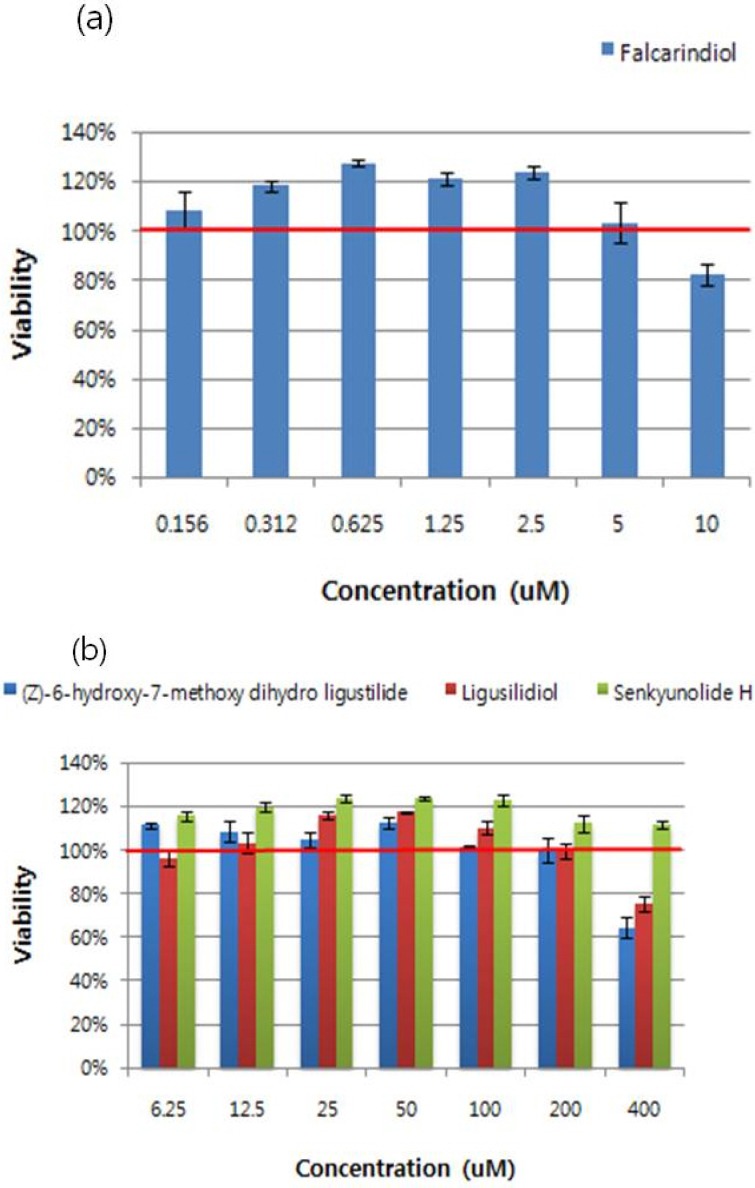
Cell cytotoxicity of RAW 264.7 cells by treatment of compounds. An examination of the cytotoxicity of four compounds on RAW 264.7 cells was evaluated by the MTT assay. Measuring cytotoxic effect each experiment was performed in triplicates and repeated at least three times. (**a**) Falcarindiol; (**b**) (*Z*)-6-hydroxy-7-methoxy-dihydroligustilide, Ligusilidiol, Senkyunolide H.

The cell cytotoxicity of compounds from *C. officinale* was assessed using a MTT assay. The cell survival values are expressed as a percentage of control treated cells. As shown in Figure 2, no significant inhibition effects on cell survival were obtained with the tested concentrations of falcarindiol (0.156~5 μM) and (*Z*)-6-hydroxy-7-methoxydihydroligustilide, ligusilidiol, senkyunolide H (6.25~200 μM) (Figure 2). Therefore, cells were treated with the indicated concentrations of compounds in the followed experiments, respectively.

#### 2.2.2. Effects of Compounds on NO Production in LPS-Stimulated RAW 264.7 Cells

**Figure 3:**
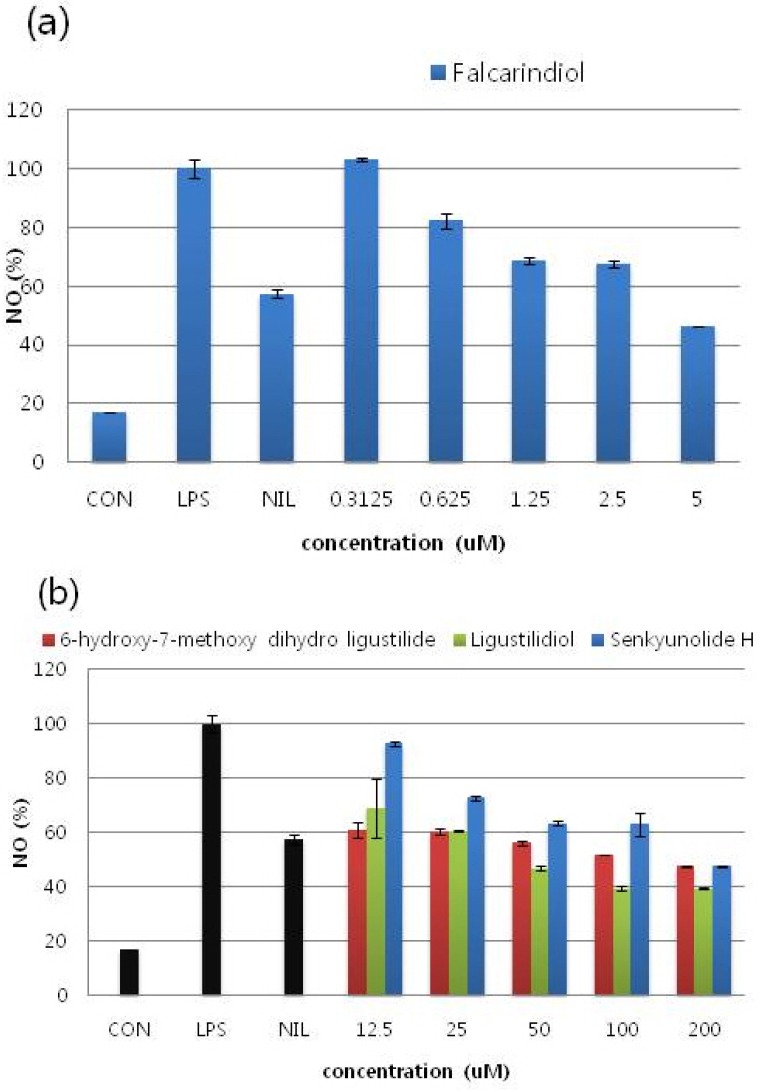
The effects of each compound with various concentrations on NO production. It was determined by measuring the level of nitrite accumulation in culture media. Among these compounds, falcarindiol showed the highest inhibition percentage for the release of NO from RAW 264.7 macrophage cells. Measuring nitric oxide accumulation each experiment was performed in triplicates and repeated at least three times. (**a**) Falcarindiol; (**b**) (*Z*)-6-hydroxy-7-methoxy-dihydroligustilide, Ligusilidiol, Senkyunolide H.

The effects of each compound on NO production was determined by measuring the level of nitrite accumulation in culture media. NO inhibitory effects of the isolated compounds on RAW 264.7 macrophage cells showed a dose dependent manner with an IC_50_ value of 4.31, 152.95, 72.78, and 173.42 μM, respectively (Figure 3, Table 2). Among these compounds, falcarindiol showed the highest inhibition percentage for the No release from RAW 264.7 macrophage cells with IC_50_ value 4.31 ± 5.22 μM (Figure 3a). Comparison with the cell viability data by MTT assay, the inhibitory effects were not attributable to cytotoxic effects. L-NIL (L-N6-(1-iminoethyl)lysine) was used as a positive control at 10 μM.

**Table 2:** IC_50_ values of compounds for LPS-induced NO production.

Treatment	IC_50_ (µM) *
Falcarindiol	4.31 ± 5.22
6-hydroxy-7-methoxy-dihydroligustilide	152.95 ± 4.23
Ligustilidiol	72.78 ±5.13
Senkyunolide H	173.42 ± 3.22
NIL	8.70 ± 2.34

* IC_50_ values were calculated from regression lines using five different concentrations. Data represent the mean ± S.D. (standard division) of triplicate experiments.

#### 2.2.3. RT-PCR for Anti-Inflammatory Effects

**Figure 4:**
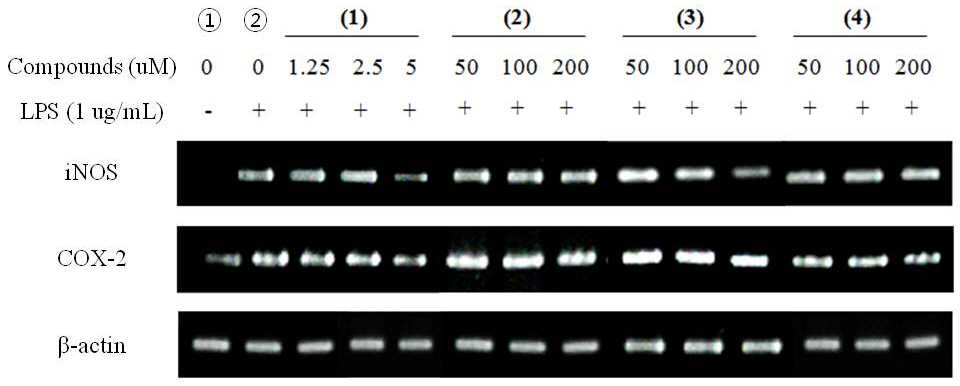
RT-PCR for anti-inflammatory effects with iNOS, and COX-2 mRNA expression in LPS-stimulated RAW 264.7 cells. The amount of RNA loaded in each lane was confirmed by β-actin mRNA. (**1**) Falcarindiol; (**2**) (*Z*)-6-hydroxy-7-methoxy-dihydroligustilide (**3**) Ligusilidiol (**4**) Senkyunolide H.

RT-PCR for anti-inflammatory effects was performed to identify the modulation of the expressions of iNOS and COX-2. In unstimulated RAW 264.7 cells, iNOS and COX-2 mRNA were not detected (Figure 4-①) and after treatment with LPS, and without pre-treatment of compounds upregulates iNOS, COX-2 expression has been detected (Figure 4-②). On the other hand, treatment of LPS with pre-treatment of compounds showed significant inhibited of corresponding upregulations without influence on the expression of β-actin, the housekeeping gene. These results indicated that the inhibitory effects of compounds on LPS-induced NO production were caused by iNOS and COX-2 suppression. Furthermore, the electrophoresis analysis of RT-PCR showed that compounds reduced mRNA levels of iNOS and COX-2 in a dose dependent manner in general (Figure 4).

### 2.3. Measurement of Anti-Cancer Activity

#### 2.3.1. Cytotoxic Effects of Falcarindiol

**Figure 5:**
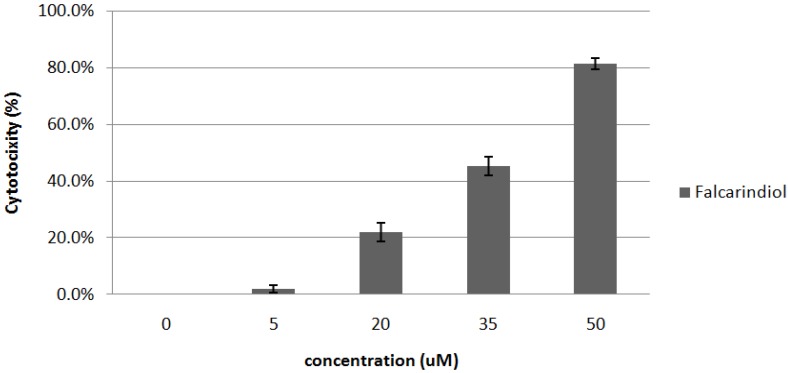
Cytotoxic effect of falcarindiol isolated from DM2 fractions of *C. officinale* on MCF-7 cells by MTT assay. Measuring cytotoxic effect each experiment was performed in triplicates and repeated at least three times.

Based on the cell cytotoxic effects by MTT assay for the isolated compounds, falcarindiol has the strongest cytotoxic effect on MCF-7 cells. As shown in Figure 5, treatment of falcarindiol with various concentrations on MCF-7 cells resulted in a marked dose-dependent cytotoxicity, with an IC_50_ value of 35.67 µM.

#### 2.3.2. Cell Cycle Assay by Flow Cytometry

**Figure 6:**
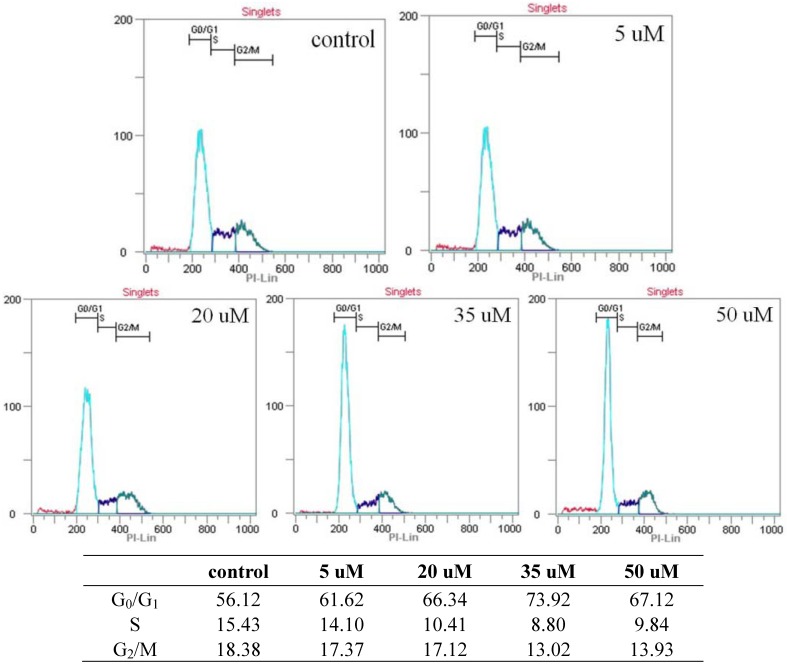
Cell cycle analysis of MCF-7 cells with treatment of falcarindiol. After 24 h of falcarindiol (5–50 µM) treatment, cells were labelled with PI and analysed by flow cytometry.

In between the concentration of 5–35 µM of falcarindiol, a significant increase of the amount of cell in G_0_/G_1_ phase was detected after 24hr (73.92% versus 56.12% in control) (Figure 6). Also, a slight increase for the amount of sub G_0_/G_1_ cells was observed in 35–50 µM of falcarindiol after 24 h. Concomitant to this increase in the amount of G_0_/G_1_ cells treated with falcarindiol, the decrease in the amount of S cells was observed in this experiment. This result suggests that the growth inhibitory effect of falcarindiol was the result of a block of cell cycle at G_0_/G_1_ phase on MCF-7 cells.

#### 2.3.3. Observation of Morphological Change

**Figure 7:**
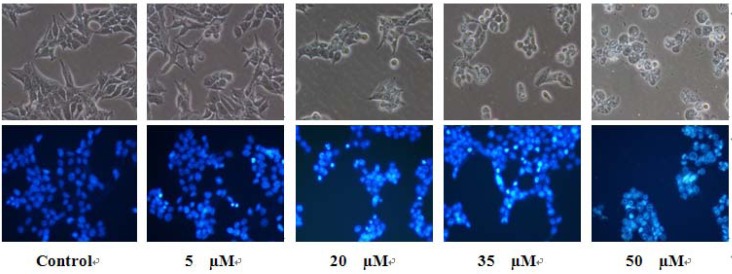
Morphological changes of MCF-7 cells observed under an inverted phase contrast microscope (400×).

The treatment of falcarindiol followed by direct observation revealed that significant morphological changes occurred on MCF-7 cells. Figure 7 showed these morphological changes of cells with falcarindiol for 24 h, the cellular morphology of MCF-7 cells was severely distorted and became round cells.

In addition, nucleic morphological changes of MCF-7 cells were observed by Hoechst 33342 staining. As shown also in Figure 7, the control cells emitted a blue fluorescence with consistent intensity, indicated that the chromatins were equivalently distributed in the nuclei. Fluorescence light of the cells with treatment of falcarindiol was turned to denser and brighter compared to untreated control cells. Also, the cells showed their chromatin condensation and karyopyknosis, which were typical apoptotic phenomena.

#### 2.3.4. RT-PCR for Anti-Cancer Effects

**Figure 8:**
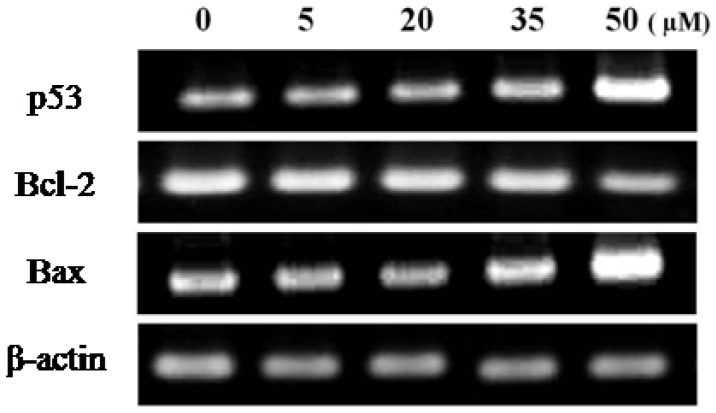
RT-PCR for anti-cancer effects on MCF-7 cells with treatment of falcarindiol.

Expression of the pro-apoptotic Bax and the anti-apoptotic Bcl-2 was examined by RT-PCR to clarify the partial mechanism of falcarindiol-induced apoptosis. The results indicated that the expression level of Bax and p53 was increased gradually, whereas that of Bcl-2 was decreased by treatment with falcarindiol on MCF-7 cells (Figure 8).

## 3. Experimental Section

### 3.1. Extraction and Isolation

#### 3.1.1. Extraction and Partition

The rhizome of *C. officinale* was purchased from Kyung-dong market, Korea, in May 2008. A voucher sample (YKCO0821) is kept in the herbarium of the College of Forest Science in Kookmin University, Seoul Korea. Dried and grinded rhizome of *C. officinale* (1.8 kg) was extracted with MeOH (8 L) three times at 46 °C. The extracts were combined and evaporated under vacuum to afford MeOH extract (251.04 g), which was partitioned between dichloromethane (DM) (1 L × 3) and water in a separatory funnel. The two layers were evaporated to obtain DM (68.84 g) and water (80.65 g) fraction.

#### 3.1.2. Isolation of Compounds

The DM fraction (68.84 g) was separated by vacuum column chromatography on silica gel (VCC, 400 mesh) column, eluting with a gradient of DM-MeOH (10:1), to give three subfractions D_1_ (44.86 g), D_2_ (12.95 g), and D_3_ (2.13 g).

The D_1_ (44.86 g) was separated by VCC, eluting with hexane-ethylacetate (H-E) (20:1), to give four subfractions D_1_-1~D_1_-4. The D_1_-2 (10.61 g) was further separated by reverse-phase silica gel column chromatography (RPC), eluting with MeOH, to afford D_1_-2-1~D_1_-2-3. The D_1_-2-1 was separated by VCC, eluting with H-E (5:1), to give compound 2 (72 mg). D_1_-2-2 was further separated by open column chromatography, eluting with H-E (1:1), to give compound 3 (60 mg).

The D_2_ (12.95 g) was separated by VCC, eluting with DM-MeOH (10:1), to give six subfractions D_2_-1~D_2_-6. The D_2_-3 was separated by VCC, eluting with H-E (1:1), to give three subfractions D_2_-3-1~D_2_-3-3. The D_2_-3-1 was further separated by RPC, eluting with MeOH-water (2:1), to afford compound **1** (94 mg).

The D_3_ (2.13 g) was separated by VCC, eluting with H-E (1:9), to give four subfractions D_3_-1~D_3_-4. The D_3_-1 was separated by RPC, eluting MeOH-water (5:1), to give compound 4 (63 mg).

#### 3.1.3. Identification of Compounds

The structures of compounds (Figure 1) were elucidated on the basis of UNIT-INOVA 300MHz NMR (Varian, USA), and mass spectral analysis with Micromass ZQ detector (Waters, Milford Massachusetts, USA). The chemical shifts are given in δ (ppm) with TMS as a internal standard and coupling constants in Hz.

*(9E)-Heptadeca-1,9-dien-4,6-diyne-3,8-diol* (**1**). ESI MS [M−H]^− ^*m/z* 259.23, C_17_H_24_O_2_, ^1^H-NMR (300 MHz, CD_3_OD) δ: 0.88 (3H, t, *J =* 6.7 Hz, H-17), 1.28 (8H, m, H-13, H-14, H-15, H-16), 1.37 (2H, m, *J =* 6.9, 7.1 Hz, H-12), 2.10 (2H, q, *J =* 6.9 Hz, H-11), 4.94 (1H, d, *J =* 5.1 Hz, H-3), 5.20 (1H, d, *J =* 8.1 Hz, H-8), 5.25 (1H, m, H-1), 5.46 (1H, m, H-1), 5.51 (1H, m, *J =* 10.5, 8.1 Hz, H-9), 5.60 (1H, m, *J =* 10.5, 6.9 Hz, H-10), 5.91 (1H, m, *J =* 17.1, 5.1 Hz, H-2); ^13^C-NMR (75 MHz, CD_3_OD) δ: 14.4 (C-17), 22.9 (C-16), 27.9 (C-11), 29.4 (C-13, C-14), 29.5 (C-12), 32.0 (C-15), 58.7 (C-8), 63.6 (C-3), 68.9 (C-6), 70.5 (C-5), 78.5 (C-4), 80.1 (C-7), 117.6 (C-1), 127.8 (C-9), 134.8 (C-10), 136.0 (C-2).

*(3Z)-3-Butylidene-4,5,6,7-tetrahydro-6-hydroxy-7-methoxy-1(3H)-isobenzofuranone* (**2**). ESI MS [M+H]^+^* m/z* 261.13, C_13_H_18_O_4_, ^1^H-NMR (300 MHz, CD_3_OD) δ: 0.87 (3H, t, *J =* 7.2 Hz, H-12), 1.42 (2H, m, *J =* 7.2, 7.5 Hz, H-11), 1.89 (2H, m, H-4), 2.27 (2H, q, *J =* 7.5, 7.8 Hz, H-10), 2.43 (2H, m, H-5), 3.45 (3H, s, -OCH_3_), 3.87 (1H, m, H-6), 4.09 (1H, d, *J =* 3.3 Hz, H-7), 5.24 (1H, t, *J =* 7.8 Hz, H-9); ^13^C-NMR (75 MHz, CD_3_OD) δ: 14.0 (C-12), 17.4 (C-4), 22.4 (C-11), 24.3 (C-5), 28.2 (C-10), 58.7 (–OCH_3_), 67.2 (C-7), 73.4 (C-6), 114.2 (C-9), 124.2 (C-8), 148.7 (C-3), 154.8 (C-2), 170.1 (C-1).

*3-Butylidene-4,5,6,7-tetrahydro-6,7-dihydroxy-1(3H)-isobenzofuranone *(**3**). ESI MS [M+Na]^+^* m/z* 247.11, C_12_H_16_O_4_, ^1^H-NMR (300 MHz, CD_3_OD) δ: 0.94 (3H, t, *J =* 7.2 Hz, H-12), 1.49 (2H, m, *J =* 7.2, 7.5 Hz, H-11), 1.99 (2H, m, H-5), 2.35 (2H, q, *J =* 7.5, 7.8 Hz, H-10), 2.56 (2H, m, H-4), 3.94 (1H, m, H-6), 4.48 (1H, d, *J=* 5.7 Hz, H-7), 5.29 (1H, t, *J =* 7.8 Hz, H-9); ^13^C-NMR (75 MHz, CD_3_OD) δ: 13.9 (C-12), 19.2 (C-4), 22.4 (C-11), 26.5 (C-5), 28.2 (C-10), 67.4 (C-7), 71.6 (C-6), 114.4 (C-9), 125.9 (C-8), 148.2 (C-3), 153.4 (C-2), 169.4 (C-1).

*(3Z)-3-Butylidene-6,7-dihydroxy-4,5,6,7-tetrahydro-2-benzofuran-1-one *(**4**). ESI MS [M+Na]^+^* m/z* 247.11, C_12_H_16_O_4_, ^1^H-NMR (300 MHz, CD_3_OD) δ: 0.88 (3H, t, *J =* 7.2 Hz, H-12), 1.42 (2H, m, *J =* 7.2, 7.5 Hz, H-11), 1.90 (2H, m, H-5), 2.27 (2H, q, *J =* 7.5, 7.8 Hz, H-10), 2.44 (2H, m, H-4), 3.39 (1H, d, *J =* 5.7 Hz, H-7), 3.90 (1H, m, H-6), 5.24 (1H, t, *J =* 7.8 Hz, H-9); ^13^C-NMR (75 Hz, CD_3_OD) δ: 14.0 (C-12), 18.9 (C-4), 22.5 (C-11), 26.2 (C-5), 28.3 (C-10), 66.8 (C-7), 71.5 (C-6), 114.4 (C-9), 125.8 (C-8), 148.3 (C-3), 154.0 (C-2), 169.7 (C-1).

### 3.2. Cell Cultures

A murine macrophage cell lines (RAW 264.7), and MCF-7 human breast cancer cell lines were purchased from the Korean Cell Line Bank (KCLB). Cell lines were cultured in Dulbecco’s modified eagle medium (DMEM) supplemented with 10% (v/v) fetal bovine serum (FBS), 100 U/mL penicillin, and 100 μg/mL streptomycin, in a humidified incubator containing 5% CO_2_ at 37 °C. Cells were treated with compounds isolated from *C. officinale* dissolved in dimethyl sulfoxide (DMSO). The final DMSO concentration in all cultures was set to 0.1%.

### 3.3. Assessment of Cell Viability

The viability of cells was assessed by the 3-(4,5-dimethylthiazol-2-yl)-2,5-diphenyltetrazolium bromide (MTT) assay according to Mosmann *et al*. [22]. Briefly, cells were seeded at 1 × 10^4^ cells/well in 96 well plates. Cells were treated with the indicated concentrations of compound 1 (0.156~10 μM) and compound 2, 3, 4 (6.25~400 μM) for 24 h. After incubation, cells were incubated with PBS contained 5 mg/mL MTT for 4hr at 37 °C in 5% CO_2_ incubator. MTT solution was then discarded and 100 μL of DMSO was added into each well to dissolve insoluble formazan crystals. Plates were then kept for 30 min at room temperature for complete solubilization. The level of colored formazan derivative was analysed on a ELISA reader (Opsys MR^TM^, Dynex) at a wavelength of 570 nm. Results were expressed as the mean percentage of cell growth inhibition. The IC_50_ value was expressed as the concentration of compounds that inhibited the growth of cells by 50%.

### 3.4. Effects of Compounds on NO Production in LPS-Stimulated RAW 264.7 Cells

To investigate the anti-inflammatory effects of isolated compounds, NO production was examined with LPS-stimulated RAW 264.7 cells by Griess reaction [23]. For NO determination, RAW 264.7 cells were seeded into a 96 well plates at a density of 1 × 10^5^ cells/well and grown for 24 h. The cells were treated with each compound for 24 h. After 24 h incubation, nitric oxide generation was determined. The 100 μL supernatants of culture media from each well were transferred to another 96 well plate and then were mixed with an equal volume of Griess reagent [1%(w/v) sulfanilamide in 5% (v/v) phosphoric acid and 0.1% (w/v) naphtylethylene-diamine-HCl]. The mixture was incubated at room temperature for 10 min, and the absorbance at 540 nm was measured using a microplate reader. Standards were prepared using a serially diluted stock solution of sodium nitrite. Measuring nitric oxide accumulation each experiment was performed in triplicates and repeated at least three times.

### 3.5. Measurement of Anti-Cancer Activity

#### 3.5.1. Cell Cycle Assay by Flow Cytometry

We used MCF-7 human breast cancer cell lines for cell cycle analysis [24]. MCF-7 cells (5 × 10^5^ cells/well) were plated into a 6 well plate and exposed to various concentrations (0, 5, 20, 35, and 50 μM) of falcarindiol for 24 h. After treatment, cells were harvest by centrifugation for 6 min at 1000 rpm. Following centrifugation the cells were fixed with 100% ethanol and stored at −20 °C. On the day of analysis, the cells were treated with 1 ml DNA staining solution (20 μg/mL propidium iodide (PI), 200 μg/mL RNase and 0.1% Triton X-100). Cells incubated in the dark at 37 °C for 30 min and analyzed with a flow cytometry (iCyt, eclipse^TM^). For each measurement, at least 10,000 cells were counted. 

#### 3.5.2. Observation of Morphological Changes

MCF-7 cells were seeded at a density of 5 × 10^5^ cells/well into a 6 well plate. After 24 h, the cells were treated with various concentration (5~50 μM) of falcarindiol. Morphological changes of cells were examined with inverted microscope (400×) at 24 h after treatment. The cells were fixed in 100% EtOH and incubated in 10 μg/mL Hoechst 33342 solution for 30 min in the dark. The cells were then examined using a fluorescence microscope (ECLIPSE Ti-U, Nikon, Japan).

### 3.6. Reverse Transcription-Polymerase Chain Reaction (RT-PCR)

Total RNAs (300 ng) of the cells treated with each compound were converted into cDNA using SuperScript II reverse transcriptase and random hexamer primers. RT-generated cDNAs encoding iNOS, COX-2 for anti-inflammatory, Bcl-2, Bax, p53 for anti-cancer, and β-actin were amplified by PCR using selective primers (Table 1). PCR amplification was performed by denaturation at 94 °C for 30 s, annealing at 55 °C for 30 s, and extension at 72 °C for 1 min for 35 cycles, and finally 72 °C for 10 min. PCR products were separated on 1% agarose gel and stained with ethidium bromide and visualized under UV light.

**Table 1:** Sequences of the primers used in RT-PCR analysis.

Genes	Primer	Sequence
iNOS	Sense	ATTGGCAACATCAGGTCGGCCATCACT
	Antisense	GCTGTGTGTCACAGAAGTCTCGAAGTC
COX-2	Sense	GGAGAGACTATCAAGATAGT
	Antisense	ATGGTGAGTAGACTTTTACA
Bcl-2	Sense	AGCTGCACCTGACGCCCTTCA
	Antisense	AGCCAGGAGAAATCACAGAGG
Bax	Sense	ATGGACGGGT CCGGGGAGCAG
	Antisense	CAGTTGAAGTTGCCGTCAGA
p53	Sense	GGGACAGCCAAGTCTGTG
	Antisense	GGAGTCTTCCA GTGTGAT
β-actin (anti-inflammatory)	Sense	TCATGAAGTGTGACGTTGACATCCGT
	Antisense	CCTAGAAGCATTTGCGGTTCACGATG
β-actin (anti-cancer)	Sense	CCTCTATGC CAACACAGTGC
	Antisense	ATACTCCTGCTTGCTGATCC

## 4. Conclusions

In this study, four compounds identified as falcarindiol (**1**), 6-hydroxy-7-methoxy-dihydroligustilide (**2**), ligustilidiol (**3**) and senkyunolide H (**4**) were isolated from a methanol extract of the dried rhizome of *C. officinale*. The three phthalides and a polyacetylene reduced the NO production for LPS-stimulated RAW 264.7 macrophage cells. Besides, falcarindiol showed a high cytotoxicity on MCF-7 cells with the lowest IC_50_ value (35.67 μM). It was found that the four compounds inhibited COX-2 and iNOS expressions in LPS-stimulated macrophage cells. This inhibitory effect probably acts at the transcriptional level, as evidenced by dose-dependent reductions in their mRNA levels. Although, inhibition of these mRNA expression was presented in LPS-stimulated RAW 264.7 cells by the four compounds, it has not cytotoxicity on RAW 264.7 cells, as assessed by MTT assay and the expression of the house-keeping gene, β-actin. It had been also demonstrated that falcarindiol indicated the cytotoxicity of breast cancer cell lines and induced apoptotic cell death with a dose dependant manner.

To determine whether the cytotoxicity of falcarindiol on MCF-7 cells involved the cell cycle arrest, we examined cell cycle phase distribution of the treated cells by flow cytometry. Data showed that falcarindiol induced a G_0_/G_1_ cell cycle block as the same trend with MTT assay results. The Hoechst 33342 staining results showed that cell shrinkage, chromatin condensation and formation of apoptotic bodies with treatment of falcarindiol (concentrations of 35, 50 μM). Also, falcarindiol induced apoptosis through strongly increased expression of Bax and p53 mRNA, and slightly reduced expression of Bcl-2 mRNA levels in a dose dependent manner. This study suggested that *C. officinale* extract and its components would be valuable candidates in the therapeutic applications as anti-inflammatory or anti-cancer agents.
